# Progress in Gynæcology

**Published:** 1900-08-18

**Authors:** 


### Progress in Gynæcology.

(*Continued from page* 320.)

**Intermenstrual Dysmenorrhœa.**—Storer,[11] writing on this subject, bases his remarks on 20 cases occurring in his own practice, adding 25 published by reliable authorities. Without coming to any definite conclusion as to origin or treatment, he finds that intermenstrual dysmenorrhœa or Mittelschmerz appeared practically every month in the whole series, except when pregnancy intervened. In 22 it always occurred on a definite day from the beginning of the last period. In 13 there was a variation of two days, and in four of four days. Thus Mittelschmerz is, as compared with normal menstruation, remarkably regular. In a large number of cases it was paroxysmal. The attacks sometimes came on at intervals of several hours, and lasted for from five to 15 minutes. In other cases there was constant pain with marked exacerbations, often of the greatest severity, and subjectively like labour pains. In about half the cases the pain resembled that of menstruation; in the rest the patients declared it was quite different. In nine cases of intermenstrual dysmenorrhœa there was no pain at the normal period. In none of the cases was there any discharge like that of menstruation. In 10 there was marked increase of leucorrhœa during the pain. Storer concludes that Mittelschmerz is a phenomenon definite in the time of its appearance, character, and seat, quite unlike premenstrual congestive pain, or the soreness that sometimes persists after menstruation, and lacking the irregularity of ovarian neuralgia. He admits that intermenstrual "dysmenorrhœa" is in no sense "dysmenorrhœa," as there is no menstrual discharge, and the pain is not like that of menstruation.

**Sclero-cystic Ovaritis.**—A. Fraikin[12] defines this disease as a chronic inflammatory affection, following usually a salpingitis, and characterised anatomically by primary sclerosis and secondary micro-cystic follicles. As regards the pathological anatomy the ovary may be in its normal situation or prolapsed: mobile or fixed by adhesions. It is always more or less enlarged, the surface is often irregular, and may be covered with fine adhesions. On section several cysts are seen, containing serum or fluid blood. The parenchyma is firm and fibrous. The Fallopian tube usually shows some lesion, which, however, may only be apparent on microscopic investigation. Discussing the ætiology, he says the affection may be met with at all ages. It is, however, especially a disease of active sexual life. Ovarian congestion is an important factor, but the principal cause is undoubtedly salpingitis. This may infect the ovary either by direct transmission through the fimbriated end, or, as is probably more frequently the case, through the lymphatics. The symptoms this disease gives rise to are various, but menstrual troubles are always present, viz., menorrhagia and dysmenorrhœa. The pain is usually felt in the ovarian region, and may radiate to the loins, down the legs, and even to the coccyx and perineum. The bimanual examination reveals a sensitive zone in the iliac region, with rigidity of the recti on pressure. The ovary is felt somewhat enlarged, and may be prolapsed or adherent. There is usually some disturbance of the general health, especially digestive troubles, palpitation, and nervous symptoms, often of a neurasthenic type. The disease must be distinguished from simple or hæmorrhagic metritis, acute or chronic salpingitis, simple prolapse of the ovary, and perimetritis. Great difficulty may be found in distinguishing the disease from "hysterical" neuralgia of the ovary, and sometimes the diagnosis can only be completed after opening the abdomen. With respect to treatment, Fraikin recommended that medical treatment, consisting of rest, tonics, hot douches, tampons, &c., should always be tried for a time. If these fail various operative measures may be undertaken. The removal of one or both ovaries has been extensively practised, but the results have been disappointing in a considerable proportion of cases. In many instances the pain is relieved for a time, and then returns as badly as ever; and, further, to this are superadded nervous phenomena of a neurasthenic type; and, although these post operative nervous troubles may be partially relieved by the administration of ovarian tabloids, the results are often unsatisfactory. On this account certain operators (Martin, Pozzi, &c.) have devised conservative operations, such as partial resection, ignipuncture, &c. They have the advantages that the organ remains functionally complete, the patient avoids the troubles of the artificial menopause, and she may become pregnant.

[11] Boston Med. and Surg. Jour., April 19, 1900. [12] Annales de Gynæc., April, 1900.

